# Timing of chronic hepatitis B diagnosis after migration and its determinants among Sub-Saharan African migrants living in France

**DOI:** 10.1371/journal.pone.0189196

**Published:** 2017-12-28

**Authors:** Julie Pannetier, Virginie Gigonzac, Nathalie Lydié, Annabel Desgrées du Loû, Rosemary Dray Spira

**Affiliations:** 1 CEPED, IRD, Université Paris Descartes, INSERM, équipe SAGESUD, Paris, France; 2 Department of social epidemiology, Institut Pierre Louis d'Epidémiologie et de Santé Publique (IPLESP UMRS1136), UPMC Univ Paris 06, Sorbonne University, INSERM, Paris, France; 3 Santé publique France, Saint-Maurice, France; University of North Carolina at Chapel Hill School of Medicine, UNITED STATES

## Abstract

**Objective:**

In European countries, chronic hepatitis B (CHB) disproportionately affects migrants from medium- and high-endemic areas and is largely underdiagnosed. To inform policy and improve screening strategies, we measured the timing of CHB diagnosis after migration and its determinants among sub-Saharan migrants living in the Paris metropolitan area (France).

**Design:**

The PARCOURS study is a retrospective life-event history survey conducted in health care services in 2012–2013 among 779 migrants from sub-Saharan Africa who were receiving care for CHB. We investigated the timing of CHB diagnosis from the time of arrival in France using the Kaplan-Meier method and characteristics associated with CHB diagnosis since the time of arrival in France using discrete-time multivariate logistic regression models.

**Results:**

The median CHB diagnosis occurred during the fourth year spent in France for men and during the second year spent in France for women. Among men, the probability of CHB diagnosis increased during years with (versus without) a temporary resident permit (aOR: 1.6, 95%CI: 1.1–2.2), a precarious accommodation (aOR: 1.7, 95%CI: 1.1–2.6), and hospitalization (aOR: 7.7, 95%CI: 3.4–15.1). Among women, CHB diagnosis was more likely to occur during years with unemployment (aOR: 1.9, 95%CI: 1.1–3.94), pregnancy (aOR: 6.6, 95%CI: 3.5–12.5) and hospitalization (aOR: 9.0, 95%CI: 2.95–32.3). For both sexes, the probability of CHB diagnosis was higher among those who migrated to France because they were threatened in their country.

**Conclusion:**

This study shows that social hardships (residential, economic, administrative) and contact with the health care system after arrival in France hasten access to a CHB diagnosis.

## Introduction

Chronic hepatitis B (CHB) remains a public health issue because of to the severity of chronic liver-related diseases (e.g., liver cirrhosis and liver cancer). Early diagnosis and treatment can reduce morbidity and mortality [1] as well as transmission to relatives through vaccination [2].

In European countries, CHB disproportionately affects migrants from medium- or high-endemic areas and is an important issue for public health policy [3–7]. People born in medium- and high-endemic countries are frequently CHB-infected during the perinatal period or within the first years of their life, which exposes them to a higher risk of chronicity [8–10]. Hence, CHB screening among migrants from endemic countries is recommended in European countries [11], including France [12]. However, testing, treatment and vaccination of migrants is not optimal because CHB screening is not systematically proposed by health professionals [6,13,14]. Barriers to CHB testing among migrants also include a fear of stigma [15] and a lack of awareness of and misconceptions about CHB [16,17].

Sub-Saharan African (SSA) migrants represented 13% of immigrants in France and 1% of the French population in 2012. They mostly originated from west and central Africa, and 60% of them live in the Paris metropolitan area (INSEE, 2012). The prevalence of CHB was estimated to be 5.25% in this population in 2004, 8 times more than in the general population (0.65%) [5]. The prevalence was also higher among SSA migrants with precarious situations [18]. Furthermore, in 2004, 80% of SSA migrants in France were believed to have ignored their hepatitis B status [5].

To inform policy and improve screening strategies, we measured the determinants and the timing of CHB diagnosis after migration among SSA migrants who were receiving care for CHB and who were living in the Paris metropolitan area using data from a large life-event survey.

## Materials and methods

### ANRS-PARCOURS study

The PARCOURS study was conducted to analyse how health trajectories and social and migratory paths were interlaced for migrants from sub-Saharan Africa living in France. This retrospective quantitative life-event survey was conducted from February 2012 to May 2013 in health-care facilities in the greater Paris metropolitan area (Ile-de-France) among three groups of migrants born in sub-Saharan Africa: one group who received HIV care, one group with CHB (without concomitant HIV infection), and a third group of people who had visited from primary-care centres. This analysis focused on the group living with CHB. Preliminary investigations were conducted to identify health centres that delivered care for CHB to sub-Saharan African migrants before a random stratified sampling was conducted. The recruitment of CHB patients occurred in 20 health care centres: 7 referenced centres for hepatitis care, 10 hospital services not referenced for hepatitis care, 2 networks of general physicians active in the field of viral hepatitis and the health centre of the Medical Committee for Exiles (Comede). To build a sample that reflected the contribution of different types of CHB care facilities in Ile-de-France, the number of individuals included from each facility was determined according to their weight within the total population of patients from sub-Saharan Africa who were followed for CHB in Ile-de-France.

In each participating facility, all outpatients aged 18 to 59 years who were born in a sub-Saharan country and had a sub-Saharan nationality at birth, were diagnosed as HBsAg+ more than three months ago and were not co-infected with HIV, were eligible.

Physicians invited all eligible patients, except those with major cognitive or health impairments, to participate and collected their written consent. Professional interpreters were available on demand. Among a total of 1169 eligible outpatients, 34 were excluded, and the participation rate among the remaining patients was 69%, for a total of 779 participants. Among the participants, detailed information on migration history, socioeconomic conditions, sexual activity and health over the patient’s lifetime was anonymously collected through a standardized life-event questionnaire administered face-to-face by a trained professional interviewer independent from the clinic staff. To ensure confidentiality, the interview occurred in a clinic setting in a private room. Each dimension of interest was documented for each year from birth until the time of data collection. Clinical and laboratory information were documented from medical records that were available in the health service where the survey was completed. Basic data about non-participants were collected anonymously. There were no major differences between participants and non-participants in demographic or clinical characteristics. The participation rate was higher among men than women (69.2% versus 61.7%, p = 0.01) and among the unemployed compared to the employed (73.0% versus 64.8%, p = 0.01) but did not differ by age or the level of transaminases.

Participants received a voucher for 15 Euros. The study was approved by the French National Commission for Data Protection and Liberties (CNIL, decision DR-2011–484). The complete survey protocol is registered at Clinicaltrials.gov (NCT02566148, https://clinicaltrials.gov/ct2/show/NCT02566148).

### Variables of interest

The date of CHB diagnosis was documented based on both the participant’s reports and the medical records. These two sources of information provided concordant dates of diagnosis (within a range of 1 year) in more than three-quarters of the cases. In the case of discordance (i.e., difference of >1 year), we considered the date of CHB diagnosis reported by the patient as the date of diagnosis in the main analysis. Alternatively, the date of diagnosis provided in the medical record was considered in a sensitivity analysis.

Covariates included fixed and time-dependent variables. Fixed covariates were educational level (elementary/secondary/higher), region of birth (West Africa, Central/Southern/Eastern Africa), reasons for coming to France (to join family, to work or study/because threatened in the country of origin/ for medical reasons) and chronic comorbidity at the time of the interview as indicated by treatment for diabetes, hypertension or cardiovascular disease. Time-dependent covariates included individual living conditions in France, which were documented annually from the year of arrival in France: housing conditions (stable/precarious), main source of income (professional activity/social benefits), employment (yes/no), stable partnership (yes/no), and residence permit (none/1 or 3 years’ temporary residence permit/long-term residence permit or French National). In addition, information on the existence of health coverage, pregnancy (either own pregnancy for women or partner’s pregnancy for men) and hospitalization was available for each year. We also considered age (<30 years/30-34 years/≥35 years), and the time that had passed since the patient’s arrival in France (1 year/2-5 years/6-10 years/>10 years) as time-dependent covariates.

### Statistical analysis

Analyses were restricted to participants who had not yet been diagnosed with CHB at the time of their arrival in France. Because CHB screening has been recommended for individuals originating from highly endemic countries in France since 2001, we additionally restricted the study population to participants who were diagnosed from 2001 onwards to consider a homogeneous time period regarding the CHB testing policy.

The probability of being diagnosed with CHB each year from the year of arrival in France was estimated using the Kaplan-Meier method. Then, characteristics associated with the probability of being diagnosed with CHB each year since the patient’s arrival in France were analysed using discrete-time multivariate logistic regression models that were adjusted for the time since the patient’s arrival in France. Covariates statistically significant at the threshold of a p-value of 0.25 in univariate analysis were introduced in the adjusted models.

Considering potential differences in CHB testing practises and determinants between men and women, all analyses were stratified by sex. Data were weighted to account for differences in the individuals’ probability of inclusion in the survey. All statistical analyses were performed using STATA 12.1 (Stata Corporation, College Station, TX).

## Results

### Characteristics of study participants

Overall, among the 779 participants of the PARCOURS survey living with CHB, 145 had been diagnosed with CHB prior to their arrival in France or before 2001. Thus, a total of 634 participants diagnosed with CHB after their arrival in France or diagnosed between 2001 and 2012 were included in this study. The recruitment strategy led to the following sample: over 52% of participant were followed in hepatology reference centres, nearly 40% in hospital services, 5.2% by the COMEDE and 2.7% by general physicians. Major characteristics of participants at the time of the interview are shown in Table 1. Participants were predominantly men (73.7%), natives of West Africa, particularly from Mali (men: 29%; women: 19%), Côte d’Ivoire (men: 16%; women: 12%) and Senegal (men: 12.5%; women: 18%), and came to France mainly for family, employment or educational reasons. The median age at the time of the interview was 38 years (IQR: 32–44) for men and 36 years (IQR: 30–41) for women, and the median length of stay in France was 10 years (IQR: 4–13) for men and 8 years (IQR: 3–11) for women. Since their arrival in France, almost one-third of participants had lived at least one year with precarious accommodations, and most had experienced at least one year of unemployment (62% of men, 84% of women) and one year without a residence permit (73% of men, 56% of women). Almost a quarter of participants had been hospitalized at least once, and two-thirds of the women and 55% of the men’s partners had been pregnant. At the time of the interview, approximately 9% of participants had a chronic comorbidity in addition to CHB infection.

**Table 1:** Characteristics of CHB-infected participants at the time of interview, by sex. ANRS-PARCOURS study.

	Men (N = 466)	Women (N = 168)
***Sociodemographics***		
**Median age (years)**a.	38	36
**Educational level (%)**a.		
**Never been to school/elementary school**	35.3	22.9
**Secondary**	39.4	60.3
**Higher education**	25.4	16.8
***Migration history***		
**Median time (years) since arrival in France**	10	7
**Region of birth (%)**		
**West Africa**	80.5	73.3
**Central/Southern/Eastern Africa**	19.5	26.7
**Reasons for coming to France (%)**		
**Family/work/study**	75.4	76.4
**Being threatened in their country**	19.7	16.8
**Medical reasons**	4.9	6.9
***Experienced at least one year from the time of arrival in France***:		
**Precarious accommodation (%)**	33.3	32.6
**Absence of financial resource (%)**	7.9	11.3
**Unemployment (%)**	62.4	84.4
**Absence of stable partnership (%)**	69.9	59.0
**Absence of residence permit (%)**	72.9	55.6
**Temporary residence permit (%)**	70.7	69.9
***Experienced since the time of arrival in France***:		
**Pregnancy** b. **(%)**	55.9	64.1
**Hospitalization (%)**	24.7	22.8
**Absence of health coverage (%)**	47.7	31.1
***Chronic comorbidity* (diabetes, hypertension or cardiovascular disease)** a.	8.9	9.3

Weighted %.

^*a*.^ At the time of interview.

^*b*.^ Among men, pregnancy of the female partner.

### Timing and circumstances of CHB diagnosis

**Fig 1:**
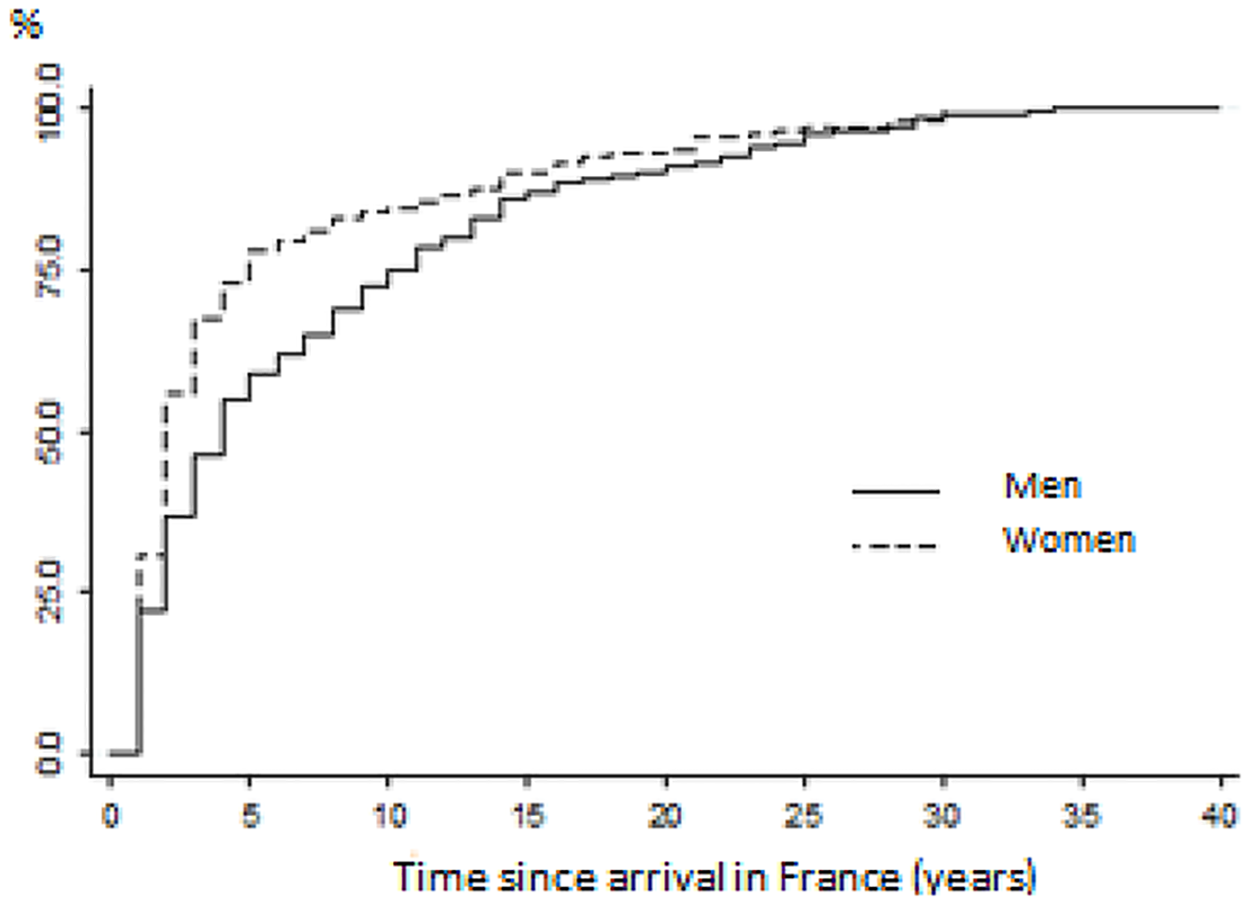
Kaplan-Meier estimate of the probability of being diagnosed with chronic hepatitis B, by gender. Parcours study, 2012–2013.

The median CHB diagnosis occurred during the fourth year spent in France for men (IQR: 2–11) and during the second year spent in France for women (IQR: 1–5) (Fig 1). Among 32% of men, CHB diagnosis was performed through systematic screening (prenatal or prenuptial testing, health check, blood donation). Nearly 27% of men reported having been tested because of a health problem that resulted from hospitalization or illness and 20% had been tested at a physician’s request. Most of the women (62%) had been diagnosed through systematic screening, mainly during prenatal testing (27% of women overall). Thirteen percent of women had been diagnosed because of a health problem and 12% had been tested at the physician’s request. Moreover, diagnosis through voluntary testing was reported by only 7% of women and 12% of men, mainly because they “wanted to know.”

### Characteristics associated with CHB diagnosis

Results of the univariate and multivariate analyses in men and women are shown in Tables 2 and 3. In the multivariate analysis, legal status was independently associated with the probability of being diagnosed after each year from the time of arrival in France, with a higher odds ratio for men who had a temporary residence permit than for those with a long term residence permit or those who were French nationals (adjusted OR: 1.6, 95%CI: 1.1–2.2). In addition, men without a residence permit tended to be diagnosed earlier than those with a long residence permit or men who were French nationals (adjusted OR: 1.3, 95%CI: 0.9–1.9). The other characteristics that were significantly associated with a higher probability of being diagnosed after each year from the time of arrival in France in the multivariate analysis included precarious accommodation (adjusted OR: 1.7, 95%CI: 1.1–2.6), having migrated to France because of being threatened in the country of origin or for medical reasons (adjusted OR: 1.8, 95%CI: 1.0–3.1), and hospitalization (adjusted OR: 7.7, 95%CI: 3.4–15.1). Being under 30 years old, having no health coverage, chronic comorbidities and an increasing duration since the arrival in France were associated with a decreased probability of being diagnosed after each year from the time of arrival in France.

**Table 2:** Characteristics associated with CHB diagnosis among men (Total PYR = 1997). ANRS-PARCOURS study. Discrete time logistic regression model.

	Men		
		Crude	Adjusted‡
	Nb of Diagnosis (PYR)	OR (95%CI)	OR (95%CI)
**Age**^1^			
**<30 years**	153 (702)	0.8 (0.7–1.1)	0.6 (0.4–0.8)†
**30–34 years**	105 (454)	0.8 (0.6–1.1)	0.7 (0.5–1.1)
**≥35 years**	208 (841)	1.0	1.0
**Educational level**			
**Elementary**	160 (640)	1.0	1.0
**Secondary**	182 (805)	0.9 (0.7–1.2)	1.0 (0.7–1.3)
**Higher education**	124 (552)	0.9 (0.7–1.2)	0.9 (0.6–1.2)
**Region of birth**			
**West Africa**	374 (1590)	1.0	1.0
**Central/Southern/Eastern Africa**	92 (407)	0.9 (0.7–1.1)	0.9 (0.7–1.2)
**Reasons for coming to France**			
**Family/work/study**	347 (1651)	1.0	1.0
**Being threatened in the country of origin**	94 (281)	1.8 (1.3–2.5)†	1.5 (1.1–2.0)†
**Medical reasons**	25 (65)	2.5 (1.3–4.9)†	1.8 (1.0–3.1)†
**Accommodation**^1^			
**Stable**	376 (1757)	1.0	1.0
**Precarious**	90 (240)	1.9 (1.3–2.8) †	1.7 (1.1–2.6)†
**Main sources of income**^1^			
**Professional activity**	410 (1834)	1.0	1.0
**Social benefits**	56 (163)	1.7 (1.1–2.7)†	1.2 (0.7–1.9)
**Employment**^1^			
**Yes**	315 (1525)	1.0	1.0
**No**	151 (472)	1.8 (1.4–2.4)†	1.2 (0.7–1.7)
**Stable partnership**^1^			
**Yes**	273 (1220)	1.0	1.0
**No**	193 (777)	1.2 (0.9–1.5)	1.2 (0.9–1.6)
**Legal status**^1^			
**Long residence permit/French national**	104 (571)	1.0	1.0
**No residence permit**	209 (895)	1.3 (1.0–1.7)†	1.3 (0.9–1.9)
**Temporary residence permit**	153 (531)	1.8 (1.4–2.5)†	1.6 (1.1–2.2)†
**Health coverage**^1^			
**Yes**	404 (1568)	1.0	1.0
**No**	62 (429)	0.4 (0.3–0.6)†	0.3 (0.2–0.5)†
**Pregnancy**^a^^1^			
**No**	411 (1788)	1.0	
**Yes**	55 (209)	1.2 (0.8–1.7)	-
**Hospitalization**^1^			
**No**	428 (1938)	1.0	1.0
**Yes**	38 (59)	8.6 (4.5–16.3)†	7.7 (3.4–15.1)†
**Chronic comorbidity**^b^			
**No**	422 (1742)	1.0	1.0
**Yes**	44 (255)	0.7 (0.5–0.9)†	0.6 (0.4–0.8)†
**Time from arrival in France**^1^			
**1 year**	108 (320)	1.0	1.0
**2–5 years**	170 (698)	0.7 (0.5–0.9)†	0.7 (0.5–1.0)
**6–10 years**	77 (419)	0.4 (0.3–0.6)†	0.4 (0.3–0.7)†
**>10 years**	111 (560)	0.5 (0.4–0.7)†	0.6 (0.3–0.9)†

^‡^Adjusted Odds Ratio (95% Confidence Interval) adjusted for age, education, region of birth, reasons for coming to France, accommodation, main sources of income, employment, stable partnership, legal status, health coverage, hospitalization, chronic comorbidities and the time from arrival in France.

PYR: person-years at risk.

^a^ Pregnancy of the female partner.

^b^At the time of interview.

^1^Time-varying covariates.

^†^ p<0.050.

**Table 3:** Characteristics associated with CHB diagnosis among women (Total PYR = 513). ANRS-PARCOURS study. Discrete time logistic regression model.

	Women		
	No. Observation with	Crude	Adjusted‡
	Diagnosis (total no.)	OR (95%CI)	OR (95%CI)
**Age**^1^			
**<30 years**	76 (261)	0.8 (0.4–1.4)	0.5 (0.3–0.9)†
**30–34 years**	45 (127)	0.9 (0.5–1.6)	0.7 (0.4–1.2)
**≥35 years**	47 (125)	1.0	1.0
**Educational level**			
**Never been to school/elementary school**	35 (124)	1.0	1.0
**Secondary**	102 (284)	1.8 (1.1–3.1)†	1.4 (0.9–2.3)
**Higher education**	31 (105)	1.6 (0.8–3.4)	1.2 (0.6–2.5)
**Region of birth**			
**West Africa**	118 (379)	1.0	1.0
**Central/Southern/Eastern Africa**	50 (134)	1.4 (0.8–2.5)	1.0 (0.6–1.8)
**Reasons for coming to France**			
**Family/work/study**	130 (447)	1.0	1.0
**Being threatened in their country**	29 (46)	5.2 (2.6–10.0)†	3.6 (1.5–9.4)†
**Medical reasons**	9 (20)	1.6 (0.8–3.5)	0.6 (0.3–1.3)
**Accommodation**^1^			
**Stable**	136 (438)	1.0	1.0
**Precarious**	32 (75)	1.6 (0.8–3.1)	0.8 (0.4–1.6)
**Main sources of income**^1^			
**Professional activity**	126 (402)	1.0	1.0
**No resources apart from social benefits**	42 (111)	1.5 (0.8–2.8)	1.3 (0.6–2.6)
**Stable or irregular employment**^1^			
**Yes**	69 (265)	1.0	1.0
**No**	99 (248)	2.2 (1.5–3.2)†	1.9 (1.1–3.4)†
**Stable partnership**^1^			
**Yes**	105 (333)	1.0	
**No**	63 (180)	1.2 (0.7–2.1)	-
**Legal status**^1^			
**Long residence permit/French national**	47 (201)	1.0	1.0
**No residence permit**	62 (176)	1.9 (1.2–3.1)†	1.4 (0.8–2.6)
**Temporary residence permit**	59 (136)	3.5 (2.1–6.0)†	1.8 (0.9–3.6)
**Health coverage**^1^			
**Yes**	150 (441)	1.0	
**No**	18 (72)	0.7 (0.4–1.3)	-
**Pregnancy**^1^			
**No**	117 (436)	1.0	1.0
**Yes**	51 (77)	4.9 (2.6–9.5)†	6.6 (3.5–12.5)†
**Hospitalization**^1^			
**No**	151 (490)	1.0	1.0
**Yes**	17 (23)	7.7 (2.3–20.0)†	9.0 (2.5–32.3)†
**Chronic comorbidities**^b^			
**No**	152 (451)	1.0	
**Yes**	16 (62)	0.7 (0.4–1.4)	-
**Time from arrival in France**^1^			
**1 year**	53 (126)	1.0	1.0
**2–5 years**	75 (186)	1.1 (0.6–2.0)	1.4 (0.8–2.6)
**6–10 years**	14 (69)	0.3 (0.1–0.7)†	0.6 (0.2–1.5)
**>10 years**	26 (132)	0.4 (0.2–0.6)†	0.6 (0.3–1.3)

^‡^Adjusted Odds Ratio (95% Confidence Interval) adjusted for age, education, region of birth, reasons for coming to France, accommodation, main sources of income, employment, pregnancy, hospitalization and time from arrival in France.

PYR = person-years at risk.

^b^At the time of interview.

^1^Time-varying covariate.

^†^ p<0.050.

In the multivariate analysis, a higher odds ratio was observed for women who had a temporary residence permit compared to those with a long residence permit or those who were French nationals. The other characteristics significantly associated with a higher probability of being diagnosed each year after the time of arrival in France in the multivariate analysis included being unemployed (adjusted OR: 1.9, 95%CI: 1.1–3.94), having migrated to France because of being threatened in the country of origin (adjusted OR: 3.6, 95%CI: 1.5–9.4), pregnancy (adjusted OR: 6.6, 95%CI: 3.5–12.5) and hospitalization (adjusted OR: 9.0, 95%CI: 2.95–32.3). Being under 30 years old was associated with a decreased probability of being diagnosed after each year from the time of arrival in France.

## Discussion

The retrospective life-event history design of our study enabled us to estimate the delay between arrival in France and CHB diagnosis after migration among sub-Saharan migrants who were receiving care for CHB. Our study shows that this delay was rather short, particularly for women. The median CHB diagnosis occurred during the fourth year spent in France for men and during the second year spent in France for women. After 5 years spent in France, 4 out of every 10 men with CHB remained undiagnosed, versus only 2 out of every 10 women. This specific design also allowed us to investigate the determinants of the diagnosis with a dynamic approach and to consider how social and health trajectories after migration were related to access to a CHB diagnosis. This study shows that social hardships (residential, economic, administrative) and contact with the health care system after arrival in France hastened the access to CHB diagnosis.

### Migrants in precarious situations have better access to CHB diagnosis than settled migrants

We have previously shown that sub-Saharan migrants often experienced social hardships (residential, economic, administrative) after their arrival in France, which exposed them to sexual risks [19]. Here, we observe that years of unemployment or of residential and administrative instability are positively related to CHB diagnosis in France. This result is consistent with a French qualitative study showing that health professionals working in structures for precarious people (mostly migrants) propose more systematic CHB screening for migrants than health professionals working in other health structures because they have a better knowledge of risk factors associated with CHB infections [13]. In particular, they are more aware of the high prevalence of CHB in the countries of origin for Asian and African migrants. This result is also consistent with a French quantitative study conducted among general physicians that showed that physicians who had a significant proportion of resource-limited patients with universal health coverage proposed more frequent CHB screening for people from high-endemic countries compared with other general physicians [20].

We also observed that CHB diagnosis was delayed in long-term established migrants in France, who were less precarious [21] and were less likely to visit health services that were dedicated to precarious people and where hepatitis screening and diagnosis was more systematically offered to migrants. This result is consistent with a study showing that migration to France that occurred over 5 years previously was associated with a lack of access to CHB screening [14].

Finally, having a chronic disease delayed access to CHB diagnosis for men. This finding is consistent with studies reporting that health professionals following men for a chronic disease did not systematically propose hepatitis screening among people known to be at risk because of their countries of origin [13,22]. Hence, except for the health services dedicated to migrants in precarious situations, the health attendants in France appear underinformed about the epidemiology of CHB, and they miss opportunities for CHB screening when they care for patients who are African migrants.

### Gender differences in CHB screening

We observed a gender difference in access to a diagnosis of CHB, with women benefiting from earlier access to CHB diagnosis than men.

For women, this phenomenon was mainly due to earlier access to the health care system through antenatal care because CHB prenatal testing has been mandatory since 1992.

For men, the time between arrival in France and the first contact with the health care system can be long, and can contribute to delayed diagnosis and care management. Similar gender imbalances have been observed regarding access to HIV diagnosis and care. Women have earlier access because they have more contact with the health care system, especially through prevention of mother-to-child transmission (PMTCT), where HIV testing is more likely to be proposed to them than to their partner [23,24].

The VESPA 2 study was a representative French survey conducted in 2011 among HIV-infected hospital outpatients that showed that among SSA migrants, the median HIV diagnosis occurred 2 years after the time of arrival in France for men and after 1 year for women [25]. At that time, HIV diagnosis occurred more rapidly than CHB diagnosis among sub-Saharan migrants, which revealed that health professionals were more informed of the risks for HIV infection in this population than of the risks for chronic CHB. However, characteristics associated with CHB and HIV diagnosis appear similar. For both HIV and CHB patients, the probability of being diagnosed after arrival was related to their social situations and their opportunities to have contact with the health care system (*Limoussy in revision*).

### Absence of CHB diagnosis may indicate a lack of contact with the health care system

Our study showed that contact with the health care system, especially through antenatal care or hospitalization, was associated with a quicker access to CHB diagnosis. Prenatal testing for CHB during antenatal care has been systematic in France since 1992, thus facilitating access to CHB diagnosis for women. Hospitalization also appears to be an opportunity for CHB screening in African migrants. In contrast, factors delaying access to CHB diagnosis indicate an overall lack of contact with the health care system. First, young people under 30 years old, who may be less likely to have contact with the health care system, were less diagnosed than older people, which was also shown in another study among the general population in France [26].

Second, having no health coverage was related to a delayed access to a CHB diagnosis. This result reinforces that one of the first barriers regarding access to care is a lack of health coverage [27,28]. In France, migrants with or without resident permit, can access health insurance coverage. In the PARCOURS study, we showed that access to health coverage for SSA migrants in France occurred within the year of their arrival but may be impaired by the lack of a residency permit [29]. Therefore, lacking a residency permit may have competing viewpoints: on one hand, it may be a barrier to access of the heath structure; on the other hand, undocumented migrants who have access to the specific health structure for migrants are more frequently offered testing than settled migrants. It should be noted that due to the nature of the data that we used, a decisive interpretation of associations in terms of causality cannot be made.

Finally, this study focused on CHB diagnosis, regardless of subsequent engagement in CHB care. However, we have previously reported among participants of the Parcours Study diagnosed with CHB in France, the large majority (93.5%) accessed to CHB care with the year following CHB diagnosis [30].

### Limits

Several limitations need to be addressed. First, the study population was restricted to SSA migrants who were consulted in hepatitis care services. We tried to recruit patients from private physicians but it was very difficult and we recruited very few. Consequently, SSA migrants with more comfortable situations may be underrepresented. In addition, information on individuals who were not receiving care for their CHB was totally missing. Nevertheless, the study was conducted among a large and diverse background group of migrants who were receiving care for their CHB infection. Second, retrospective cross-sectional studies are subjected to memorization bias, although the life-event questionnaire improved the recall effort and the consistency of collected information [31]. Third, that the data were self-reported can also be one of limitations of this study, but we found a strong consistency between the date of diagnosis reported by the patient and the date of diagnosis reported by the physician. Furthermore, the results remained unchanged with sensitivity analyses that were conducted and considered the date of diagnosis provided by the physician.

## Conclusion

This article provides useful results to help improve access to CHB screening among migrants from SSA living in France, who are heavily affected by this infection. Our results suggest that screening strategies should be expanded to include migrants that have negligible contact with social and health services and young people and migrants who migrated to France in the past. Screening of CHB should be proposed in primary care settings and outside the health care system. General practitioners should more systematically propose a CHB screening to all their patients from SSA. Also, as was the case for HIV, CHB screening can be improved with the development of community screening that uses rapid tests [6,32,33].

## Key messages

Women benefiting from an earlier access to CHB diagnosis than men.

The median CHB diagnosis occurred during the fourth year spent in France for men and during the second year spent in France for women.

Migrants in precarious situations have better access to CHB diagnosis than settled migrants

Absence of CHB diagnosis may indicate a lack of contact with the health care system
